# Deep learning in bioinformatics

**DOI:** 10.55730/1300-0152.2671

**Published:** 2023-12-18

**Authors:** Malik YOUSEF, Jens ALLMER

**Affiliations:** 1 Department of Information Systems, Zefat Academic College, Zefat, Israel; 2 Medical Informatics and Bioinformatics, Institute for Measurement Engineering and Sensor Technology, Hochschule Ruhr West, University of Applied Sciences, Mülheim an der Ruhr, Germany

**Keywords:** Deep learning, bioinformatics, neural networks, biological data analysis

## Abstract

Deep learning is a powerful machine learning technique that can learn from large amounts of data using multiple layers of artificial neural networks. This paper reviews some applications of deep learning in bioinformatics, a field that deals with analyzing and interpreting biological data. We first introduce the basic concepts of deep learning and then survey the recent advances and challenges of applying deep learning to various bioinformatics problems, such as genome sequencing, gene expression analysis, protein structure prediction, drug discovery, and disease diagnosis. We also discuss future directions and opportunities for deep learning in bioinformatics. We aim to provide an overview of deep learning so that bioinformaticians applying deep learning models can consider all critical technical and ethical aspects. Thus, our target audience is biomedical informatics researchers who use deep learning models for inference. This review will inspire more bioinformatics researchers to adopt deep-learning methods for their research questions while considering fairness, potential biases, explainability, and accountability.

## 1. Introduction

**Figure 1:**
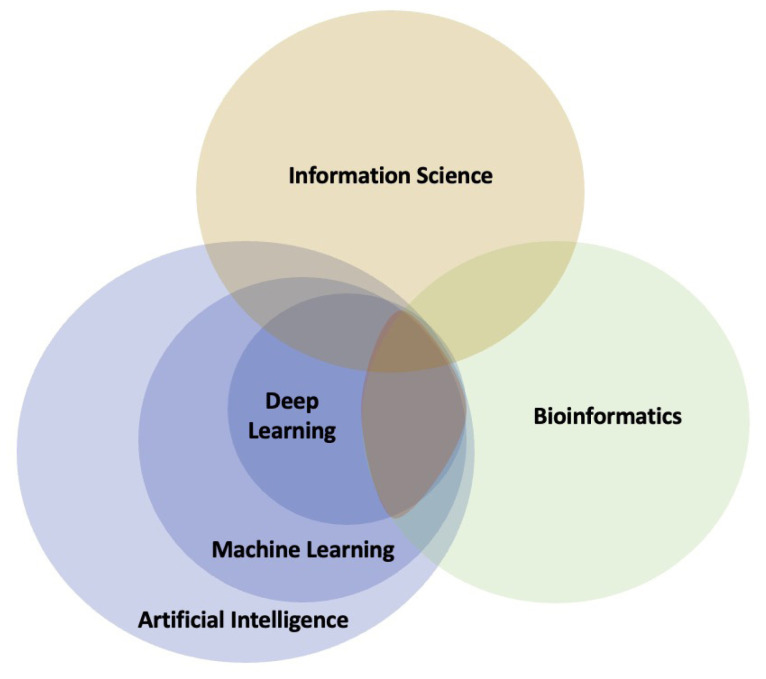
Relationships among bioinformatics, information science, artificial intelligence, machine learning, and deep learning. At the intersection of all circles (orange) is the application of AI, ML, and DL in other areas, such as bioinformatics.

Biology has become a data-driven science, for example, due to next-generation sequencing and mass spectrometry (Schadt et al. 2010). Precision medicine will depend on big data because patient stratification needs to be informed, which is best done through omics analyses (Twilt, 2016; Hulsen et al., 2019). Data analysis in these fields is performed by bioinformatics and medical informatics (Chen et al., 2017). Big data, however, is not enough; it needs to be turned into information and knowledge (Mahoto et al., 2021). Therefore, the following sections explore the intersections of information science, artificial intelligence, and other fields (Figure 1).

Information science is a field of study concerned with collecting, organizing, analyzing, interpreting, and disseminating information (Seadle and Havelka, 2023). It encompasses a broad range of topics, including library science, information management, and data science, and has applications in fields such as education, business, and government (Cervone, 2016).

Artificial intelligence (AI) is the simulation of human intelligence in machines designed to think and act like humans (Korteling et al., 2021). AI systems can be trained to perform various tasks, such as image and speech recognition, decision-making, and language translation (Litjens et al., 2017). AI has become increasingly prevalent in society, with applications ranging from healthcare to autonomous vehicles (Bohr and Memarzadeh, 2020). Furthermore, AI has been used to develop novel algorithms and insights that have been used to improve many existing processes (Cordero et al., 2019; Tran et al., 2019).

Machine learning (ML) is a subfield of AI that involves the development of algorithms and statistical models that enable computers to “learn” from data (Yazdani et al., 2023). Machine learning aims to allow computers to make predictions or take actions based on input data without being explicitly programmed to do so (Baştanlar and Ozuysal, 2014). In bioinformatics, machine learning has been used to make predictions regarding the protein structure (AlQuraishi, 2021), protein-protein interactions (Sarkar and Saha 2019), gene expression (Al taweraqi and King, 2022), and disease diagnosis (Ahsan et al., 2022).

Deep learning (DL) is a subfield of machine learning based on artificial neural networks inspired by the structure and function of the human brain (Lepakshi, 2022). Deep-learning algorithms consist of multiple layers of interconnected nodes, each performing a specific computation on the data it receives (LeCun et al., 2015). The use of deep learning and the availability of enormous computing resources has revolutionized the field of AI, allowing computers to achieve human-like accuracy in tasks such as image and speech recognition (Hosny et al., 2018).

Deep-learning algorithms have proven highly effective in various tasks, such as image and speech recognition, natural language processing, and autonomous decision-making. For example, deep-learning algorithms have been used to classify images from the ImageNet dataset field accurately (Yang et al., 2020). Deep-learning algorithms have been used in speech recognition to achieve near-human-level accuracy (Litjens et al., 2017). Natural language processing also uses deep-learning algorithms to generate human-like text (Davenport and Kalakota, 2019). In autonomous decision-making, deep-learning algorithms have been used to control robotic agents (Sarker et al., 2021). The ability of deep-learning algorithms to automatically learn complex representations from raw data has led to remarkable breakthroughs in artificial intelligence (Alzubaidi et al., 2021).

In summary, although all these fields are related, they differ in focus and scope. Information Science deals with managing and disseminating information, whereas AI and ML focus on developing algorithms that enable computers to simulate human intelligence. Deep learning is a specific machine learning approach based on artificial neural networks.

In this study, we discuss some arbitrarily selected DL applications in bioinformatics to showcase the general impact of DL on bioinformatics. In addition, we raise the questions that users of such DL models should ask when assessing them for use in their research. We aim to provide a high-level yet comprehensive overview of the issues that may arise when applying DL models in bioinformatics. The work covers questions ranging from considering the original training data to making DL models somewhat more interpretable. We hope that equipped with these questions, bioinformaticians who employ DL models for inference can perform proper model selection.

In the following sections, ML, DL, and the relevance of DL in bioinformatics will be detailed.

### 1.1. Machine learning

Machine learning is a subfield of artificial intelligence that involves developing algorithms that can learn patterns in data and make predictions or decisions based on the trained model. Machine learning is used in many applications, from image recognition and natural language processing to autonomous vehicles and medical diagnosis (Acosta et al., 2022).

One of the fundamental requirements for machine learning is numeric data. Machine learning algorithms require numeric data because they use mathematical models to make predictions or decisions. Numeric data can be represented as a matrix, where each row represents a sample and each column represents a feature. Features are the characteristics of the data that the algorithm uses to make predictions or decisions.

In some cases, the available data may be in a different form than the numeric data. In these cases, the data must be transformed into numeric data before it can be used with machine learning algorithms. This process is called feature engineering and involves selecting, transforming, and combining the features in the data to create a new set of numeric features that can be used with machine learning algorithms (Roe et al., 2020).

In addition to numeric data, machine learning algorithms require high-quality data. High-quality data are accurate, complete, and representative of the problem the algorithm is trying to solve. High-quality data is essential because it ensures that the algorithm learns the correct patterns so that the resulting model can make accurate predictions or decisions (Habehh and Gohel, 2021).

Expert-crafted features can also be necessary for machine learning algorithms. Expert-crafted features are numerical representations of the data designed by domain experts who understand the problem the algorithm is trying to solve. Expert-crafted features can help improve machine learning algorithms’ performance by providing more relevant information for the algorithm to learn from, especially if the amount of training data is restrictive (Lin et al., 2020).

Different machine learning algorithms can be used, depending on the problem that needs to be solved. Some popular learning algorithms include the following (Hastie et al., 2009; Jovel and Greiner, 2021):

- Linear regression is an algorithm that attempts to find the best-fit line that describes the relationship among variables.
- Logistic regression is a classification algorithm that predicts the probability of an event occurring.
- Decision trees are hierarchical algorithms that use a series of binary decisions to make predictions.
- Random forest is an ensemble algorithm that combines multiple decision trees to improve performance.
- Support vector machines are a classification algorithm that attempts to find the best hyperplane that separates the data into different classes.
- Neural networks represent an algorithm that uses layers of interconnected nodes to learn complex patterns from the data.

In conclusion, machine learning is a powerful tool that can solve many problems. It requires numeric data, high-quality data, and sometimes expert-crafted features. There are different learning algorithms to choose from depending on the problem that needs to be solved.

### 1.2. Deep learning

Neural networks were introduced decades ago. Back then, they typically consisted of a few layers (input, hidden, and output) of neurons, with the self-organizing maps being the smallest and containing only two layers (input and processing/presentation). Following the large increase in computational power since then, a larger number of layers can be handled today, and with more hidden layers, the networks are referred to as deep neural networks. Typically, deep learning models (deep neural networks) consist of three main layers: the input, hidden, and output layers.

**Figure 2:**
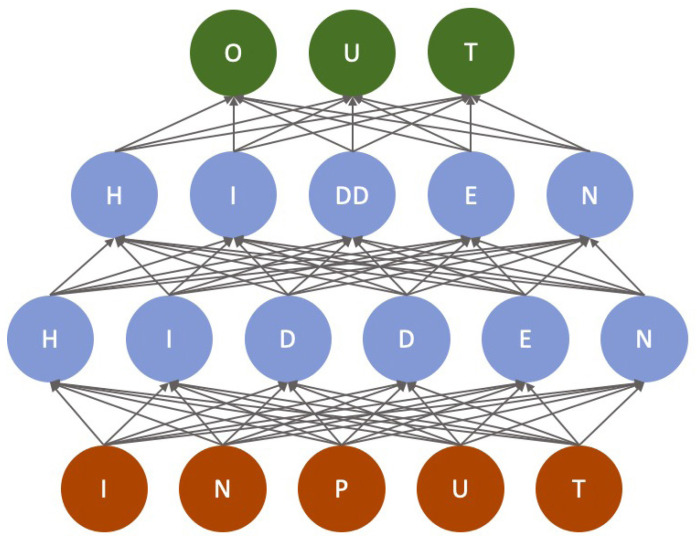
Fully connected deep learning network. The neurons of the input layer are in orange, those of the hidden layers are in blue, and the neurons of the output layer are in green. The connections in this example are directed from input to output and are indicated by arrows. Each arrow represents a trainable weight.

The input layer (Figure 2, orange nodes) is the starting point of the neural network. It receives raw data or features used as input for the deep learning model. Each feature or data point corresponds to a node in the input layer. The values of these nodes are passed forward through the network for further processing. The number of neurons in the input layer is governed by the input. For example, if the input is a coin toss, one neuron would be enough to represent that. The more complex the input, the more neurons are needed.

Hidden layers (Figure 2, blue nodes) are intermediate between the input and output layers. They are responsible for transforming input data into more abstract and meaningful representations. Deep learning models can have multiple hidden layers, making them “deep” networks. Each node in a hidden layer can be connected to every node in the previous and subsequent layers; however, other connectivity, such as convolutional layers, is also used. The connections between nodes are represented by weights, adjusted during training to optimize the model’s performance. The hidden layers can have any number of neurons, and there is no clear rule regarding how many neurons there should be. This needs to be established for each DL problem. The hidden layers also do not need the same number of neurons; they may increase, decrease, or remain the same throughout the hidden layers. In our example (Figure 2), the hidden layers contain more neurons than the input layer, but this is not always true.

The output layer is the final layer of the deep learning model. It produces the desired output or prediction based on the information processed in the hidden layers. Therefore, the number of nodes in the output layer depends on the specific task for which the model is designed. For example, in a classification task, each node in the output layer may represent a different class. In contrast, a single node may represent the predicted numeric value in a regression task. If the result is binary, one node would suffice.

Through training, deep learning models are trained by adjusting the weights in the connections between nodes to minimize errors and improve their performance on a specific task. This training is often accomplished using large numbers of labeled data and optimization techniques such as backpropagation and gradient descent. When developing a DL model, it is typical that different architectures of the DL network are tried and that the most suitable architecture for the task is chosen on empirical grounds.

The major difference between deep learning and traditional machine learning is that DL detects higher-level features automatically. In contrast, ML depends on features suggested or generated by experts in the field (Sarker, 2021). Automatically discerning higher-level features can lead to a significant drawback in explainability, which is vital in healthcare, where it is crucial to understand the decision-making process (the Precise4Q consortium et al., 2020). For example, a decision tree transparently discloses all steps from input to decision. At the same time, a DL model implicitly contains all the information leading to the decision but needs to be explored or understood by humans.

We illustrate the major difference between traditional machine learning and deep learning by focusing on two aspects: feature generation and detection, as well as the interpretability of the models (Table).

On the left side, we represent traditional ML, where features are suggested or generated by experts in the field. This highlights the involvement of domain knowledge and human expertise in identifying the relevant features of the model. These features serve as inputs to the ML model, which then goes through the decision-making process. The decision-making process can be represented as a simple flow, such as a decision tree, where each step is easily understandable and traceable. This emphasizes the explainability and transparency of traditional ML models.

On the right side, we depict deep learning, which excels at automatically detecting higher-level features from raw data. Table represents this by showing an automatic feature detection process. DL models consist of complex, layered neural networks that can learn and extract abstract representations and features from data without explicit human intervention. However, this complexity also contributes to the need for more transparency and interpretability. While the DL model implicitly contains all the information leading to a decision, understanding the decision-making process or exploring the reasoning behind it is more complex for humans.

### 1.3. Why deep learning is relevant to bioinformatics

Bioinformatics involves the application of computational methods to analyze biological data (Bayat, 2002). Bioinformatics uses machine learning to analyze and interpret biological data. However, traditional machine learning methods require manual curation of features, which can be challenging. On the other hand, deep learning can learn higher-level features directly from the data (Lee et al., 2018). If it is possible to derive a mathematical formula that describes a problem, machine learning is no longer needed to address the issue. However, biology is a complex interplay of many factors that cannot be modeled in its entirety using mathematical formulas. Hence, the application of machine learning, specifically deep learning, is warranted. For example, in gene expression analysis, traditional machine learning methods involve the manual curation of features (Monaco et al., 2021). While this is relatively easy for gene expression, predicting whether an RNA sequence is a pre-microRNA is more challenging. Thousands of features need to be manually curated, and it needs to be clarified whether all of these features are relevant (Sacar and Allmer, 2013). In such cases, DL can learn higher-level features directly from the data (Kim et al., 2016), making it highly relevant to bioinformatics.

Despite the promising results achieved by deep learning in bioinformatics, challenges still need to be addressed. One of the major challenges is the need for large amounts of high-quality data (Sarker, 2021; Son et al., 2022). Deep-learning models require large amounts of data to learn complex patterns, and the data quality directly impacts the model’s accuracy. Another challenge is the interpretability of deep-learning models (Meng et al., 2022). Deep-learning models are often described as “black boxes” since it is difficult to understand how they arrive at their predictions (Azodi et al., 2020).

In the following section, we will first discuss some applications of deep learning in bioinformatics before exploring ways to help shed light on the decision-making process of the so-called black boxes.

## 2. Selected applications of deep learning in bioinformatics

We have selected the following eight deep-learning tools because they represent a diverse range of applications in bioinformatics, demonstrate the power and flexibility of deep-learning approaches, and showcase the benefits of integrating deep learning into various aspects of biological research. The selected tools are DeepBind (Alipanahi et al., 2015), DeepCpG (Angermueller et al., 2017), DeepGene (Yuan et al., 2016), DeepFam (Seo et al., 2018), DeepLoc (Thumuluri et al., 2022), DeepPath (Coudray et al., 2018), ScanNet (Tubiana et al., 2022), and DeepVariant (Poplin et al., 2018). We will provide a short description of each tool in the following sections. All these tools can be considered black boxes, which limits the interpretability of their predictions (we discuss interpretability in section 3.3). Additionally, all models are trained on a subset of the chemical/biological possibilities so that they can have potential issues with the generalizability of the model. On the other hand, overfitting is a possibility for these models, which also hinders the generalizability of the model. We discuss these issues in section 3.1. Apart from these and other challenges, mentioned elsewhere in this text, we point out some limitations with each of the tools.

DeepBind (Alipanahi et al., 2015) is a deep-learning tool that predicts the binding specificity of DNA- and RNA-binding proteins and the effects of genetic mutations on these interactions. Using convolutional neural networks, DeepBind can identify binding patterns from large datasets of sequence information. This tool has significantly improved the accuracy of predicting protein-DNA interactions and has broad applications in understanding gene regulation and the impact of noncoding genomic variants in diseases. DeepBind has its limitations; for example, binding sites are heterogeneous (e.g., size, location, and sequence composition), which puts into question whether DeepBind can accurately predict all these types. Additionally, binding is a dynamic process that is further influenced by the environment, such as salt concentration. It is unlikely that all these influences are accurately modeled.

DeepCpG (Angermueller et al., 2017) is a deep-learning model that predicts methylation states in single cells using bisulfite sequencing data. By employing a combination of convolutional and recurrent neural networks, DeepCpG accurately models spatial and long-range dependencies in methylation patterns, providing valuable insights into the epigenetic landscape of individual cells. This tool is helpful for studying development, cellular differentiation, and diseases associated with epigenetic changes, such as cancer. DeepCpG is primarily designed for single-cell methylation data and, therefore, performance may degrade when using it for cell populations. Also, CpGs are not evenly distributed throughout a genome, and methylation patterns can vary across cell types. These limitations should be considered when using DeepCpG.

DeepGene (Yuan et al., 2016) is a deep-learning-based classifier for cancer subtypes using somatic point mutations. It employs a combination of restricted Boltzmann machines and deep neural networks to learn hierarchical representations of mutational patterns. This approach enables accurate classification of cancer subtypes and reveals potential driver mutations that contribute to carcinogenesis, providing valuable insights for precision oncology and personalized medicine. While many genes are well-studied across species, DeepGene was trained on a specific dataset, which may not include all possibilities. Additionally, genetic sequence features are very heterogenic, which may further limit the generalization of the model.

DeepFam (Seo et al., 2018) is a protein family classification tool that uses deep learning to predict the functional family of a given protein sequence. By employing a 1D convolutional neural network (CNN), DeepFam captures local and global sequence features, resulting in highly accurate family predictions. This tool aids in the functional annotation of proteins and supports large-scale analyses of protein sequence datasets, facilitating the discovery of novel protein families and studying protein evolution. When using the tool, one should keep in mind that protein families, for example, transcription factors, are highly heterogeneous and that many noncanonical protein sequences exist that may pose challenges to DeepFam.

DeepLoc (Thumuluri et al., 2022) is a deep-learning-based tool for predicting the subcellular localization of proteins. Using a combination of convolutional and recurrent neural networks, DeepLoc captures both sequence-based and evolutionary information, resulting in highly accurate localization predictions. This tool is essential for understanding protein function, protein-protein interactions, and cellular processes in various organisms. Similar to DeepBind, the environmental conditions exert a strong influence on prediction accuracy, which can be exemplified by membrane proteins. Especially with posttranslational modifications, this is a formidable challenge. Another limitation is that many proteins exist in multiple locales and that not always a confidence measure is attached to the predictions.

DeepPath (Coudray et al., 2018) is a deep-learning approach for inferring gene regulatory networks using gene expression data. By employing a combination of unsupervised feature learning and supervised classification, DeepPath learns the regulatory relationships between genes, providing insights into the complex regulatory mechanisms that govern cellular processes. This tool has broad applications in the study of gene regulation, disease mechanisms, and the development of therapeutic interventions. DeepPath may be sensitive to sequence variations, which may hamper the recognition of binding sites. Additionally, missing indirect evidence for binding or noncanonical sequence can limit the performance of DeepPath.

ScanNet (Tubiana et al., 2022) employs a geometric deep-learning model that directly learns features from protein structures to predict functional sites such as binding sites for small molecules, other proteins, or antibodies. ScanNet is accurate, versatile, and interpretable, making it suitable for functional site prediction tasks. It effectively detects protein-protein and protein-antibody binding sites and predicts epitopes of the SARS-CoV-2 spike protein. The same limitations that apply to DeepBind also apply to ScanNet. The applicability of ScanNet to noncanonical sequences may be especially limited.

DeepVariant (Poplin et al., 2018) is a deep-learning-based approach for variant calling in high-throughput sequencing data. Employing a deep neural network, DeepVariant identifies genomic variants with high accuracy and sensitivity while reducing false-positive calls. This tool is essential for studying genetic variation in populations, understanding the genetic basis of diseases, and advancing personalized medicine efforts. Data quality, especially base-calling accuracy, together with the depth of sequencing, affects DeepVariant’s effectiveness. The same is true for contaminations in the sequencing data from, e.g., microbes.

In summary, these eight deep-learning tools showcase the versatility and power of deep-learning approaches in tackling diverse bioinformatics challenges. By harnessing the power of deep learning, these tools have significantly advanced our understanding of complex biological processes and contributed to various applications, including functional annotation, protein design, disease mechanism investigation, and personalized medicine. As deep-learning techniques continue to evolve and improve, they will undoubtedly play an increasingly important role in advancing the field of bioinformatics and our understanding of the underlying principles governing life. Clearly, all tools have general limitations that apply to all DL tools and have specific limitations, as pointed out above. However, tools that are not based on DL may have similar and/or further limitations, and we believe that with DL, predictions have become more accurate.

Bioinformatics encompasses many topics, tools, and analytical approaches. Deep learning is an approach that can be applied to many such areas. In the following, we will briefly mention some areas and indicate the selected tools. Keep in mind, that the same general limitations mentioned above and detailed later in the text apply to all the tools mentioned.

### 2.1. DNA sequencing

#### 2.1.1. Sequence assembly

The genome of a species must be available for many downstream bioinformatics tasks. Therefore, sequence assembly is one of the first tasks performed in bioinformatics for any species of interest. Many tools for genome assembly exist and have been compared in an Assemblathon (Bradnam and Fass, 2013). Interestingly, we could not find an assembly tool that employs deep learning. However, for the assembly of metagenomes, MetaVelvet-DL (Liang and Sakakibara, 2021) is an extension of MetaVelvet (Namiki et al., 2012) that incorporates deep learning. The original MetaVelvet algorithm is an extension of the Velvet assembler, optimized explicitly for metagenomic data. It works by constructing de Bruijn graphs from the input sequencing reads and then identifying and partitioning these graphs into individual species or subgraphs to assemble the genomes of individual organisms. MetaVelvet-DL improves upon the original MetaVelvet algorithm by incorporating deep-learning techniques to better handle the complexity and diversity of metagenomic data. Using deep neural networks, MetaVelvet-DL can more accurately identify and partition the de Bruijn graphs, improving genome assembly and better resolution of individual species within the microbial community.

#### 2.1.2. Genome annotation

The next step in bioinformatics analysis following sequence assembly is genome annotation.

The previously mentioned DeepVariant can be employed to annotate variants in a genome. Other approaches that are more directly targeted to genome annotation have been proposed (Yip et al., 2013; Shen et al., 2022). A more complete solution for this purpose is presented by DeepAnnotator (Amin et al., 2018), which provides a generalized computational approach for genome annotation at an F-score of 94%. DeepAnnotator is a deep-learning-based tool for functional annotation of proteins. It employs deep neural networks to predict protein function by classifying protein sequences into functional categories based on their amino acid sequences. The main goal of DeepAnnotator is to assign functional roles to proteins, which is essential for understanding the biological processes occurring within cells and for studying protein interactions, pathways, and the impact of genetic variations on protein function. DeepAnnotator uses a hierarchical deep neural network architecture to capture local and long-range dependencies within protein sequences, enabling it to learn complex sequence features and patterns associated with specific protein functions. The deep-learning model is trained on large-scale protein sequence datasets with known functional annotations, enabling it to recognize and predict the functions of novel protein sequences.

#### 2.2. Gene expression analysis

Following genome assembly and annotation, another measure is the expression of such genes in an organism, especially the differential expression among different phenotypes. Several studies have shown that deep-learning models provide more accurate predictions of gene expression than traditional methods (Amin et al., 2018; Avsec et al., 2021). High-throughput gene expression profiling technologies, such as DNA microarrays and RNA sequencing, provide large gene expression datasets that can be analyzed using deep-learning algorithms (Zhang et al., 2021). Deep convolutional neural networks (CNNs) are currently the state-of-the-art method for predicting gene expression from DNA sequences (Avsec et al., 2021). Deep learning has also been used to discover biomarkers and identify genetic variations in human genomics (Alharbi and Rashid, 2022; Shen et al., 2022). Therefore, deep learning has been successfully applied to gene expression analysis in bioinformatics.

#### 2.3. Gene function prediction

Gene function prediction is similar to genome annotation but focuses on the biological roles of the identified genes. Several tools are available for gene function prediction in bioinformatics.

DeepGOPlus (Kulmanov and Hoehndorf, 2021) is a deep-learning-based tool for predicting the function of proteins using their amino acid sequences. DeepGOPlus employs a neural network architecture, a precise combination of convolutional neural networks (CNNs) and long short-term memory (LSTM) networks, to predict gene ontology (GO) terms associated with proteins. DeepGOPlus captures local and global sequence features, leading to highly accurate predictions of molecular function, biological processes, and cellular component GO terms. This tool aids in understanding protein function and supports functional annotation efforts for newly sequenced genomes.

DeepGMAP (Onimaru et al., 2020) is a deep-learning-based tool for predicting the genomic location of transcription factor binding sites (TFBSs) using ChIP-seq data. DeepGMAP employs a combination of convolutional neural networks (CNNs) and recurrent neural networks (RNNs) to model the spatial and sequence-specific patterns of TF binding. DeepGMAP significantly improves the accuracy and specificity of TFBS prediction compared with traditional approaches, providing valuable insights into gene regulatory networks and the functional impact of noncoding genetic variants.

DeepNF (Gligorijević et al., 2018) is a deep-learning-based tool for predicting protein-protein interactions and functional associations. Using an unsupervised deep-learning approach, it integrates multiple types of biological data, such as protein sequence, domain composition, and protein-protein interaction networks. DeepNF employs stacked autoencoders to learn a joint representation of the input data, which can then be used to predict protein-protein interactions and functional associations accurately. This tool is essential for studying protein function, cellular processes, and the development of therapeutic interventions targeting specific protein interactions.

DeepMir (Cordero et al., 2019) is a deep-learning-based tool for identifying and classifying microRNA (miRNA) precursors, which are small noncoding RNAs that play crucial roles in gene regulation and are implicated in various biological processes and diseases. DeepMir employs a convolutional neural network to predict miRNA precursor sequences from a given genomic sequence represented as abstract images. The model achieves high accuracy and includes initial steps toward its explainability.

The use of DL can tackle many more challenges in bioinformatics, and the tools that have been mentioned so far showcase that all areas of bioinformatics currently see the application of DL. However, the application of DL in bioinformatics is challenging. Some of these will be discussed below.

### 2.4. Protein structure prediction

Protein structure prediction is a fundamental problem in bioinformatics because the 3D structure of a protein determines its function and interactions. However, experimentally determining protein structures is costly and time-consuming, and only a fraction of known proteins have their structures solved. Therefore, developing computational methods to accurately predict protein structures from their amino acid sequences is a major challenge and a long-standing goal of bioinformatics research. This has long been realized, and a protein structure prediction challenge was created in the 1990s (Moult et al., 1997).

One of the most successful computational methods for protein structure prediction is AlphaFold 2 (Skolnick et al., 2021), a deep-learning tool developed by Google DeepMind. AlphaFold uses a novel deep-learning architecture to learn complex patterns and relationships between amino acids in the protein sequence and predict their distances and angles in 3D space. AlphaFold also incorporates evolutionary information from multiple sequence alignments and uses a graph neural network to represent the protein as a complex system of interacting amino acids. AlphaFold outputs a confidence score for each predicted structure, indicating its reliability.

AlphaFold has demonstrated remarkable performance in the Critical Assessment of Protein Structure Prediction (CASP), a biennial community challenge for testing the accuracy of protein structure prediction methods. In CASP13 (2018), AlphaFold placed first among the participating teams, showing significant improvements over previous methods (AlQuraishi, 2019). In CASP14 (2020), AlphaFold achieved an average accuracy competitive with experimental structures, effectively solving the protein structure prediction problem in most cases (Jumper et al., 2021).

### 2.5. Disease diagnostics and drug discovery

Disease diagnostics is a crucial task in bioinformatics and healthcare because it involves identifying and classifying diseases based on various data types, such as clinical symptoms, laboratory tests, medical images, and genomic sequences. Disease diagnostics can benefit from applying deep learning tools, which can learn complex patterns and features from large-scale data and make accurate and robust predictions. This area is wide-ranging from multi-omic data evaluation to the application of chatbots in anamnesis. In this overview of deep learning in bioinformatics, we cannot go into details and invite the interested readers to consider the works by Park et al., Kumar et al. and Myszczynska and colleagues (Myszczynska et al., 2020; Park et al., 2021; Kumar et al., 2023). These examples illustrate the potential and diversity of deep learning tools for disease diagnostics in bioinformatics. By applying deep learning to various types of data, these tools can improve disease diagnostics’ accuracy, efficiency, and reliability and contribute to a better understanding of disease mechanisms and outcomes.

After disease diagnosis, drug discovery is another task that is too large to be discussed in detail in this small overview. Drug discovery is a challenging and costly process that involves identifying and optimizing novel chemical entities that can modulate biological targets and treat diseases. Deep learning has seen considerable adoption in the field. Please consider the following three examples of the potential and diversity of deep learning tools for drug discovery in bioinformatics. These tools can improve the efficiency, accuracy, and creativity of drug discovery and development by applying deep learning to various types of data, such as chemical structures, protein sequences, biological assays, and clinical outcomes.

DeepChem (Altae Tran et al., 2017) is an open-source deep learning framework for drug discovery. It provides various modules and functionalities for data preprocessing, model building, model evaluation, and model deployment. DeepChem can be used for various drug discovery tasks, such as molecular property prediction, virtual screening, de novo drug design, and drug synthesis planning.

ODDT (Wójcikowski et al., 2015) is an open-source tool for computer-aided drug discovery (CADD). It integrates various methods and algorithms for molecular docking, pharmacophore modeling, similarity searching, machine learning, and deep learning. ODDT can be used for various CADD tasks, such as target identification, hit identification, lead optimization, and ADMET prediction.

Cyclica is a company that uses deep learning to accelerate drug discovery (Abdollahi et al., 2023). It offers various solutions for target identification, polypharmacology prediction, drug design, and drug repurposing.

## 3. Challenges of deep learning in bioinformatics

While information concerning data may be considered only crucial for model training, it is essential to openly disclose the data used in model training so that model users can inspect it. With this information, potential users can consider the model, compare it to others, and decide whether to use it or not.

### 3.1. Training data

Training data quality plays a crucial role in the success of deep-learning applications. High-quality data are essential for training accurate and robust deep-learning models that can effectively capture complex biological patterns and relationships (Fan and Shi, 2022). With an increase in data dimensions, more data is needed to train effective models; therefore, the quantity of the data becomes important and, with it, whether the data represents all diverse aspects of the biological phenomenon. Proper annotation and handling of noise and errors are also important. While it may seem of little importance to know how a model was trained when it reaches high accuracy, it may only do so for specific data. To judge this, information about the training data is needed, and several aspects must be considered.

#### 3.1.1. Quantity of data

Deep-learning models usually require large amounts of data for training because they can learn complex patterns and representations (Lee et al., 2022). Insufficient data regarding data and model dimensionality can lead to overfitting, where the model memorizes the training data and does not generalize well to new, unseen data (Demšar and Zupan, 2021). In bioinformatics, obtaining large-scale datasets can be challenging because of various factors, such as the cost and time associated with experimental data generation, limited availability of well-annotated data, and the inherent complexity of biological systems (Faustino et al., 2008). A simple example would be creating a daily melatonin cycle model. We would need at least hourly measurements to allow an hourly resolution of the prediction. To make it more general, we would need this for many days, not one day. We suggest having ten times more data than the model parameters for DL models. Inspecting how the model was trained can help decide whether the model is suitable for the intended purpose.

#### 3.1.2. Representation and diversity of the data

Data used for training deep-learning models should represent the studied biological system and cover various examples and scenarios (Ching et al., 2018). Bioinformatics means including data from various species, tissues, experimental conditions, and disease states. A diverse and representative dataset ensures that the model can capture the variability and complexity of biological systems and make accurate predictions on new, unseen data. When considering a DL model for inference, the amount and breadth of data used to train the model should be considered in conjunction with the purpose. If the breadth of the data that is supposed to be processed is covered in the model’s training data, it can be suitable even if it may not be suitable for another closely related dataset. However, biological data can be noisy and subject to various sources of error (Tsimring, 2014). These errors can negatively impact the performance of deep-learning models, leading to inaccurate predictions and reduced generalizability (Karimi et al., 2020).

#### 3.1.3. Noise and error

Biological data, especially those generated by high-throughput experimental techniques, can be noisy and subject to various sources of error (Li et al., 2019). These errors can arise from technical issues such as sequencing errors, experimental variability, batch effects, or other biological factors such as genetic variation or rare and uncharacterized sequences. Noisy and error-prone data can negatively impact the performance of deep-learning models, leading to inaccurate predictions and reduced generalizability (Alipanahi et al., 2015; Karimi et al., 2020). With an increasing amount of data, the impact of noise and error diminishes. Therefore, a model trained on large amounts of data in relation to the model parameters is preferable.

#### 3.1.4. Annotation quality and consistency

In supervised deep learning, models are trained on data with known labels or annotations, such as protein functions or gene regulatory relationships. The quality and consistency of these annotations directly influence the model’s performance (Chen et al., 2021). In bioinformatics, annotations can be derived from experimental data, literature curation, or computational predictions, and their quality and reliability can vary widely. Inaccurate or inconsistent annotations can lead to poor model performance and misleading predictions.

#### 3.1.5. Data preprocessing and normalization

Appropriate data preprocessing and normalization are critical for ensuring that the input data are suitable for deep-learning models (Imran et al., 2022). Some bioinformatics analyses involve various steps, such as sequence alignment, quality control, feature extraction, and data transformation. Careful preprocessing and normalization can reduce the impact of noise and errors, ensure comparability across different datasets, and improve the performance of deep-learning models. This is even more important when considering metagenomic data. An overview of preprocessing steps for preparing microbiome sequencing data for machine learning is given by (Ibrahimi et al., 2023). Normalization of sequencing results, for example, in transcriptomics, is essential, and a recent evaluation can be found here (Ni and Qin, 2021). However, in biology, there is often no gold standard data, so normalization and preprocessing approaches cannot easily be benchmarked. Additionally, simply changing some cutoff value, e.g., for counts after RNA-seq analysis, can have a large impact on the results (Beukers and Allmer, 2023).

In conclusion, data quality is critical to the success of deep-learning applications in bioinformatics. Ensuring sufficient quantity, diversity, and representation of data, minimizing noise and errors, maintaining high-quality annotations, and employing appropriate preprocessing and normalization techniques are essential for developing accurate and robust deep-learning models that can advance our understanding of complex biological systems and contribute to various applications in molecular biology, genetics, and systems biology.

### 3.2. Computational requirements

Applying deep learning in bioinformatics often demands substantial computational resources because of the complexity of biological data and the inherent computational intensity of deep-learning algorithms (Moreno et al., 2022). This is especially true when training DL models; however, the computational requirements can also be prohibitive for running already trained models for inference.

#### 3.2.1. Processing power

Deep-learning models, especially those with multiple layers, many neurons, and many edges, require significant processing power for training and inference. Inference on a laptop or PC may not be possible, depending on the DL model. High-performance processors, such as graphics processing units (GPUs) or specialized tensor processing units (TPUs), are often used to accelerate deep learning computations, as they are specifically designed for parallel processing of large-scale mathematical operations. For smaller models, it is possible to perform inference on a PC with an average GPU; however, several GPUs may be needed for larger models. Alternatively, many cloud services offer GPU access hourly so that calculations can be performed in the cloud. For example, TPUs introduced by Google are available on the Google cloud. Other large open-source projects, such as HuggingFace, also offer access to computing.

#### 3.2.2. Memory capacity

Deep-learning models require considerable memory capacity, particularly those with billions to trillions of parameters and extensive input data. The model parameters, input data, and intermediate values, such as activations and gradients, must be stored in the memory during training. Insufficient memory capacity can limit the size and complexity of models that can be trained and the size of the input data that can be processed. Thus, having sufficient RAM and GPU memory is crucial for deep learning in bioinformatics. While training is resource-intensive, inference, the process of making predictions with a trained model, also demands a large memory capacity. It is necessary to accommodate the model’s size, especially if it has been trained with several parameters. An insufficient amount of RAM during the inference phase could lead to suboptimal processing speeds, thereby affecting the usability and efficiency of the model. The number of parameters is especially prohibitive in large language models such as GPT4, but many bioinformatics applications may not be as resource-hungry. AlphaFold, for instance, has only around 100 million parameters, which is several orders of magnitude smaller than GPT4. Hence, many bioinformatics DL models may run on consumer hardware. While AlphaFold may run with 32 GB of RAM, more is better.

#### 3.2.3. Data storage

Bioinformatics datasets, especially those generated by high-throughput experimental techniques, can be enormous, necessitating substantial storage capacity. Deep-learning models often require access to large-scale training data to learn complex patterns and representations effectively. As a result, deep-learning applications in bioinformatics may require extensive storage solutions, such as high-capacity hard drives, solid-state drives, or distributed storage systems. LLMs such as GPT may need tens of TB of data to train the model, but much less data storage capacity is needed for inference. Some bioinformatics applications, such as AlphaFold, require a relatively large storage capacity. AlphaFold, for instance, stores large sequence databases and requires 3 TB of hard disk space. Faster storage is preferable. Today, some laptops, such as the recent Macbook Pro, can come with a 4 TB hard drive capacity and 64GB of RAM, allowing the execution of AlphaFold.

### 3.3. Interpretability and explainability

One of the major challenges associated with deep-learning models is their black-box status. It is often difficult to understand how the model arrives at its predictions. In bioinformatics, where the interpretation of results is critical for understanding biological processes, the need for more interpretability of deep-learning models is a significant concern (Petkovic et al., 2018). In this section, we explore the current state of interpretability in deep-learning models and discuss opportunities for overcoming the black box status of these models.

One approach is to use model-agnostic methods for interpretability. These methods involve analyzing the model’s behavior using perturbations or sensitivity analysis. Examples of such methods include Local Interpretable Model-Agnostic Explanations (LIME) and Shapley Additive exPlanations (SHAP) (Sathyan et al., 2022). LIME (Ribeiro et al., 2016) is a model-agnostic method that explains individual predictions of a deep-learning model by creating a simplified, interpretable model that approximates the behavior of the original model. LIME generates perturbed versions of the input data and measures how the model’s output changes for each perturbation. Then, LIME fits a linear or logistic regression model to the perturbed data and uses the regression model coefficients as weights to assign importance scores to each input feature. LIME can explain different data types, such as text, images, or tabular data. SHAP is another model-agnostic method based on the concept of Shapley values, which are derived from game theory and measure how much each player contributes to the outcome of a cooperative game. SHAP considers each input feature as a player and calculates the Shapley value for each feature by averaging all possible combinations of features and measuring how much the feature changes the model’s output when added or removed from the combination. SHAP can explain different models, such as tree-based models, deep neural networks, or kernel-based models.

Another approach is to use model-specific methods for interpretability. These methods involve modifying the architecture of the deep-learning model to incorporate explicit mechanisms for interpretability. Such methods include attention mechanisms, which allow the model to focus on specific parts of the input, and saliency maps, which highlight important input regions contributing to the model’s prediction (Figueroa et al., 2022). Additional layers can be incorporated into the deep-learning architecture to support the explainability (Vinuesa and Sirmacek, 2021).

Incorporating domain knowledge into the deep-learning model is another way to explain models’ decisions. In bioinformatics, domain knowledge can guide learning and constrain the model to produce biologically meaningful results. For example, in gene expression analysis, prior knowledge of gene regulatory networks can be incorporated into the deep-learning model to improve its interpretability (Fortelny and Bock, 2020). Another approach is to use a multimodal data integration (Zhu et al., 2023). In bioinformatics, multimodal data integration can involve combining data from different sources, such as gene expression data, protein-protein interaction data, and pathway data, to improve the interpretability of the deep-learning model. Integrating multiple data sources makes generating more comprehensive and biologically meaningful models possible.

Several future directions can be pursued to overcome the black box status of deep-learning models in bioinformatics (Teng et al., 2022). One approach is to develop hybrid models that combine deep learning with machine learning methods, such as rule-based systems or decision trees (Ferry et al., 2023). By combining different strategies, it may be possible to generate more interpretable models that capture the complexity of biological systems. Another direction is to develop methods for evaluating the interpretability of deep-learning models (Meng et al., 2022). Currently, there is yet to be a widely accepted metric for assessing the interpretability of deep-learning models. Developing such a metric would enable researchers to compare the interpretability of different models and create new, more interpretable models.

We explored the explainability of DL models in our study on pre-microRNA prediction using DL (Cordero et al., 2019). Because we transformed the input data into images, we could build on top of large image models. This also enabled us to explore which parts of an image support the decision of whether an image represents a pre-miRNA or not, employing saliency maps (Simonyan et al., 2013).

## 4. Ethical and social implications

Applying deep learning in bioinformatics has great potential to advance biological knowledge and improve human health. However, it also raises ethical and social issues that must be addressed and resolved. These questions are equally valid for training and inference. When using a model for inference, it is essential to ensure that the results are unbiased. Some of these issues are as follows:

### Data privacy

Deep learning requires large amounts of data to train and validate its models, which may include sensitive personal or health information. Critical ethical questions that need to be considered are how to protect the privacy and confidentiality of such data and obtain informed consent from the data providers or participants (Li et al., 2019).

### Bias

Deep learning models may inherit or amplify biases in data or algorithms, leading to unfair or inaccurate outcomes or decisions. For example, deep learning models for disease diagnosis may perform differently for different populations or subgroups, depending on the data quality and representation (Ellis et al., 2022). Detecting and mitigating such biases and ensuring the fairness and transparency of deep learning models are crucial social challenges that need to be addressed (Pagano et al., 2023).

### Accountability

Deep learning models may significantly impact the lives and well-being of individuals or society, especially when used for high-stakes applications such as drug discovery or precision medicine. However, deep learning models are often complex and opaque, making it difficult to explain or understand their logic or reasoning. How to ensure the accountability and responsibility of the developers and users of deep learning models and how to establish appropriate regulations and standards for their development and application are essential ethical and social issues that need to be resolved (Floridi et al., 2018).

## 5. Conclusion

### 5.1. Summary of deep learning in bioinformatics

Deep learning in bioinformatics refers to applying advanced neural network architectures and algorithms to analyze and interpret complex biological data. By leveraging the power of deep learning, researchers can uncover hidden patterns, relationships, and features within biological data, leading to new insights and discoveries in molecular biology, genetics, and systems biology.

Some critical aspects of the application of deep learning in bioinformatics areas follow:

- Handling of diverse biological data types. Deep-learning techniques can process various kinds of biological data, such as DNA sequences, protein sequences, gene expression data, and protein-protein interaction networks.
- Development of specialized deep-learning architectures. Customized deep-learning architectures, such as convolutional neural networks, recurrent neural networks, and autoencoders, are employed to tackle specific bioinformatics tasks, such as protein function prediction, gene regulatory network inference, and protein structure prediction.
- Quality of training data and its preprocessing. Ensuring high-quality data and appropriate preprocessing techniques are critical for the success of deep-learning applications in bioinformatics. This includes handling noise, errors, and diverse data representation.
- Computational requirements: Deep learning in bioinformatics requires substantial computational resources, such as processing power, memory capacity, data storage, network bandwidth, and scalability, to handle the complexity of biological data and the computational intensity of deep-learning algorithms.

Deep learning has significantly advanced the field of bioinformatics, enabling researchers to tackle complex challenges and gain a better understanding of biological processes. It has been applied to various bioinformatics tasks, such as functional annotation, protein design, disease mechanism investigation, and personalized medicine.

### 5.2. Future directions for deep learning in bioinformatics

Deep learning is a powerful tool for analyzing and interpreting biological data. Its ability to learn higher-level features directly from the data makes it highly relevant to bioinformatics, where traditional expert manual feature crafting approaches may be too time-consuming. Although there are challenges to be addressed, the continued application of deep learning in bioinformatics holds great promise for advancing our understanding of biological systems.

As deep learning continues to advance significantly in various fields, its application in bioinformatics is also expected to grow and evolve.

Integrating multiple omics data types, such as genomics, transcriptomics, proteomics, and metabolomics, can provide a more comprehensive understanding of biological systems. Deep-learning models can be designed to integrate and analyze multi-omics data effectively (Kang et al., 2022), leading to improved predictions, a better understanding of disease mechanisms, and the identification of novel biomarkers and therapeutic targets.

While deep-learning models have shown great success in various bioinformatics tasks, their predictions are often considered black boxes because the knowledge representation in the model is not explicit. Developing interpretable and explainable deep-learning models is essential for building trust and understanding the biological basis of their predictions, which can lead to more actionable insights and hypotheses. Developing interpretable deep-learning models will also be a key area of research, enabling us to understand these systems’ underlying biology better.

In bioinformatics, obtaining large-scale, well-annotated data can be challenging. Transfer learning, as we performed for pre-miRNA prediction, and few-shot learning approaches, which involve leveraging pretrained models or learning from small amounts of data, can be employed to overcome data limitations and improve the performance of deep-learning models in tasks with limited training data.

Developing deep-learning models that can generalize well across different biological systems, species, and experimental conditions is essential for their broad applicability. Techniques to improve model generalization and robustness, such as domain adaptation and data augmentation, can enhance the utility of deep-learning models in bioinformatics. This is an important area, as seen from our work on pre-miRNA prediction (Saçar Demirci et al., 2017) and de novo sequencing (Savas Takan and Allmer, 2023).

Biological systems exhibit complex behavior across multiple scales, ranging from the molecular to cellular, tissue, and organism levels. Developing deep-learning models capable of capturing and integrating information across different scales can lead to a more comprehensive understanding of biological processes and the relationships between different levels of organization.

As deep learning advances, there will be an increasing need for interdisciplinary collaboration among computer scientists, biologists, and other domain experts. These collaborations will facilitate the development of novel deep-learning methods tailored to the unique challenges of bioinformatics and help bridge the gap between computational predictions and biological validation.

Continued advancements in hardware, such as GPUs, TPUs, and neuromorphic chips (Pastur Romay et al., 2016), will enable the training of larger and more complex deep-learning models. Furthermore, developing efficient and scalable deep-learning software frameworks will facilitate the application of deep learning to bioinformatics challenges.

In summary, the future of deep learning in bioinformatics is expected to involve the development of novel models and techniques, improved integration of multi-omics data, enhanced interpretability, better generalization and robustness, multiscale modeling, interdisciplinary collaboration, and advancements in hardware and software. These directions will help deepen our understanding of complex biological systems, drive discoveries, and contribute to various molecular biology, genetics, and systems biology applications.

## Figures and Tables

**Table:** Differences between machine learning using traditional algorithms and machine learning using deep neural networks.

	ML	DL
**Algorithms**	Many different (SVM, DT, kNN, …)	Defined by architecture (RNN, GAN, LSTM, ...)
**Data size**	Can work well with smaller inputs	Requires large amount of data
**Performance**	Typically extremely fast	Computational complexity depends on the architecture
**Features**	Hand-crafted	Can be learned
**Preprocessing**	Significant effort	Can be trained on raw data
**Fine tuning**	Setting the algorithm parameters	Can be performed automatically during training
**Complexity**	Typical simple mathematical models	Depends on the architecture (highly flexible)
**Transparency**	Typically transparent	Hard to transparently show decision making
**Explainability**	Typically explainable	Hard to show the reasoning process
